# Comparison of Coronally Advanced Flap and Semilunar Coronally Repositioned Flap for Root Coverage of Gingival Recession in Maxillary Anterior Teeth: A Clinical Comparative Study

**DOI:** 10.7759/cureus.115274

**Published:** 2026-08-27

**Authors:** Shah Naz, Dudala Rambabu, Savan S R, Amit De, Nitubroto Biswas

**Affiliations:** 1 Periodontology, Kusum Devi Sunderlal Dugar Jain Dental College and Hospital, Kolkata, IND; 2 Periodontology, Haldia Institute of Dental Sciences and Research, Haldia, IND; 3 Periodontology, Buddha Institute of Dental Sciences and Hospital, Patna, IND

**Keywords:** clinical attachment gain, coronally advanced flap, gingival recession, root coverage, semilunar coronally repositioned flap

## Abstract

Introduction

Gingival recession is a common mucogingival condition that may result in dentinal hypersensitivity, root caries, cervical abrasion, and compromised aesthetics, particularly in the maxillary anterior region. Various root coverage procedures have been proposed for the management of gingival recession defects. Among the available treatment options, the coronally advanced flap (CAF) and semilunar coronally repositioned flap (SCRF) are relatively simple techniques for the treatment of isolated shallow recession defects.

Aim

This study aimed to compare the clinical outcomes of CAF and SCRF in the treatment of Miller's class I gingival recession defects in maxillary anterior teeth.

Materials and methods

Twenty patients presenting with isolated Miller's class I gingival recession defects in maxillary anterior teeth were enrolled in this randomized, single-blind controlled clinical study. Patients were randomly allocated into two groups (n = 10 each). Group A received treatment with CAF, whereas Group B was treated using the SCRF technique. Clinical parameters including recession height (RH), probing depth (PD), clinical attachment level (CAL), and width of keratinized gingiva (WKG) were recorded at baseline, at three months, and at six months postoperatively. Additionally, the percentage of root coverage was calculated at six months.

Results

Both treatment modalities resulted in improvement of clinical parameters. Notably, SCRF was found to be a simple, rapid, and sutureless procedure with favorable gain in the WKG. However, CAF demonstrated superior clinical outcomes with respect to percentage of root coverage, frequency of complete root coverage, and gain in CAL at six months.

Conclusion

Both CAF and SCRF were effective in the management of Miller's class I gingival recession defects. However, CAF achieved superior root coverage and attachment gain compared with SCRF, suggesting that CAF remains the preferred treatment modality for predictable root coverage in isolated maxillary anterior recession defects.

## Introduction

Gingival recession is defined as the apical migration of the gingival margin beyond the cementoenamel junction (CEJ), resulting in exposure of the root surface [1]. Clinically, it is a frequently observed condition associated with multiple etiological factors, including aggressive toothbrushing, periodontal disease, chronic inflammation, aberrant frenum attachment, and orthodontic tooth movement through a thin buccal alveolar plate. As a result, it may lead to dentinal hypersensitivity, root caries, cervical abrasion, plaque accumulation, and aesthetic concerns, particularly in the maxillary anterior region [2,3].

Root coverage procedures are a critical part of periodontal plastic surgery, intending to restore the gingival margin to its original position while improving aesthetics and reducing patient discomfort. Ideally, a successful root coverage procedure should provide predictable coverage of the exposed root surface, adequate soft tissue thickness, increased width of keratinized gingiva (WKG), and harmonious blending with the adjacent tissues [1,2].

Among the various root coverage techniques available, the CAF is one of the most widely accepted and reliable surgical techniques for the treatment of localized gingival recession defects. It is favored for its simplicity, versatility, and consistent long-term results, making it the most frequently performed procedure for root coverage [4]. In contrast, the semilunar coronally repositioned flap (SCRF), originally described by Tarnow, is a less invasive technique which is tension-free and suture less that preserves the interdental papillae, thus achieving good aesthetic integration with the surrounding tissues [5].

Although both techniques have demonstrated favorable outcomes in the treatment of shallow recession defects, comparative clinical evidence remains limited. Therefore, the present randomized controlled clinical study was conducted to evaluate the clinical outcomes of CAF and SCRF in the treatment of Miller's class I gingival recession defects in maxillary anterior teeth.

## Materials and methods

Study population and study design

This randomized, single-blind, prospective controlled clinical study was conducted in the Department of Periodontology after taking approval from the Institutional Ethics Committee. The study adhered to the ethical standards outlined in the Declaration of Helsinki (1975, revised 2000). Moreover, written informed consent was obtained from all participants after thoroughly explaining the nature, risks, and benefits of the clinical investigation and associated procedure.

The study group consisted of 20 patients presenting with isolated Miller's class I gingival recession defects in maxillary anterior teeth who were recruited from the outpatient clinic of the Department of Periodontology. The sample size was calculated using G*Power software (Heinrich-Heine-Universität Düsseldorf, Düsseldorf, Germany), which indicated a minimum of 10 participants and a maximum of 13 participants per group. To ensure consistency of the study population, specific inclusion criteria were used: adult patients aged between 20 and 45 years with no contraindications for periodontal surgery and who had not taken any medications known to interfere with periodontal tissue health or healing in the past six months, exhibiting the presence of Miller's class I gingival recession in the maxillary anterior tooth with probing depth (PD) ≤3 mm with no bleeding on probing, along with at least 2 mm of WKG in the mid-facial region of the tooth, and absence of caries or restorations in the sites to be treated. Excluded from the study were individuals with untreated periodontal disease, smokers, subjects with immunosuppressive systemic diseases, and conditions such as the presence of apical radiolucency, cervical abrasion, erosion, root caries, restorations, non-vital teeth, or teeth with periapical pathology or occlusal trauma at the study site, as well as individuals who had a previous history of lack of compliance with the maintenance therapy.

All patients underwent phase I periodontal therapy, including oral hygiene instructions, scaling and root planing, and correction of traumatic toothbrushing habits. Following initial therapy, the patients were instructed to use a soft-bristled toothbrush and maintain optimal plaque control. Each recession defect was randomly assigned to one of the two treatment groups: Group A: CAF group (n = 10) and Group B: SCRF group (n = 10). Block randomization was performed, and the allocation was recorded in a concealed allocation sheet inaccessible to the clinical examiner.

Clinical measurements

A full-mouth Plaque Index (PlI) [6] and Gingival Index (GI) [7] were recorded at baseline of the study. Clinical measurements were obtained at the mid-facial aspect of the treated teeth using a UNC-15 periodontal probe. To minimize bias, all measurements were recorded by a trained, calibrated examiner who was blinded to treatment allocation. The mucogingival junction was identified using the roll technique and visual inspection. Recession height (RH) was measured from the CEJ to the gingival margin. PD was measured from the gingival margin to the base of the sulcus. Clinical attachment level (CAL) was calculated as the sum of RH and PD. The width of the keratinized gingiva (WKG) was measured from the gingival margin to the mucogingival junction. All the measurements were recorded in millimeters. These parameters were evaluated at baseline, at three months, and at six months. To assess the treatment outcome, percentage root coverage was calculated at six months using the following formula [8]:

$\text{Root coverage (\%)} = \frac{\text{Baseline RH} - \text{6-month RH}}{\text{Baseline RH}} \times 100$

Surgical procedures

A single experienced operator performed all surgical procedures. Local anesthesia was administered using 2% lignocaine containing 1:80,000 epinephrine. After obtaining anesthesia, thorough root preparation was performed, including root planing, reducing the convexity of the root surface, and removing sharp edges and grooves. Following instrumentation, the root surfaces were washed with saline solution to remove all the debris and tissue fragments.

Coronally advanced flap procedure

**Figure 1:**
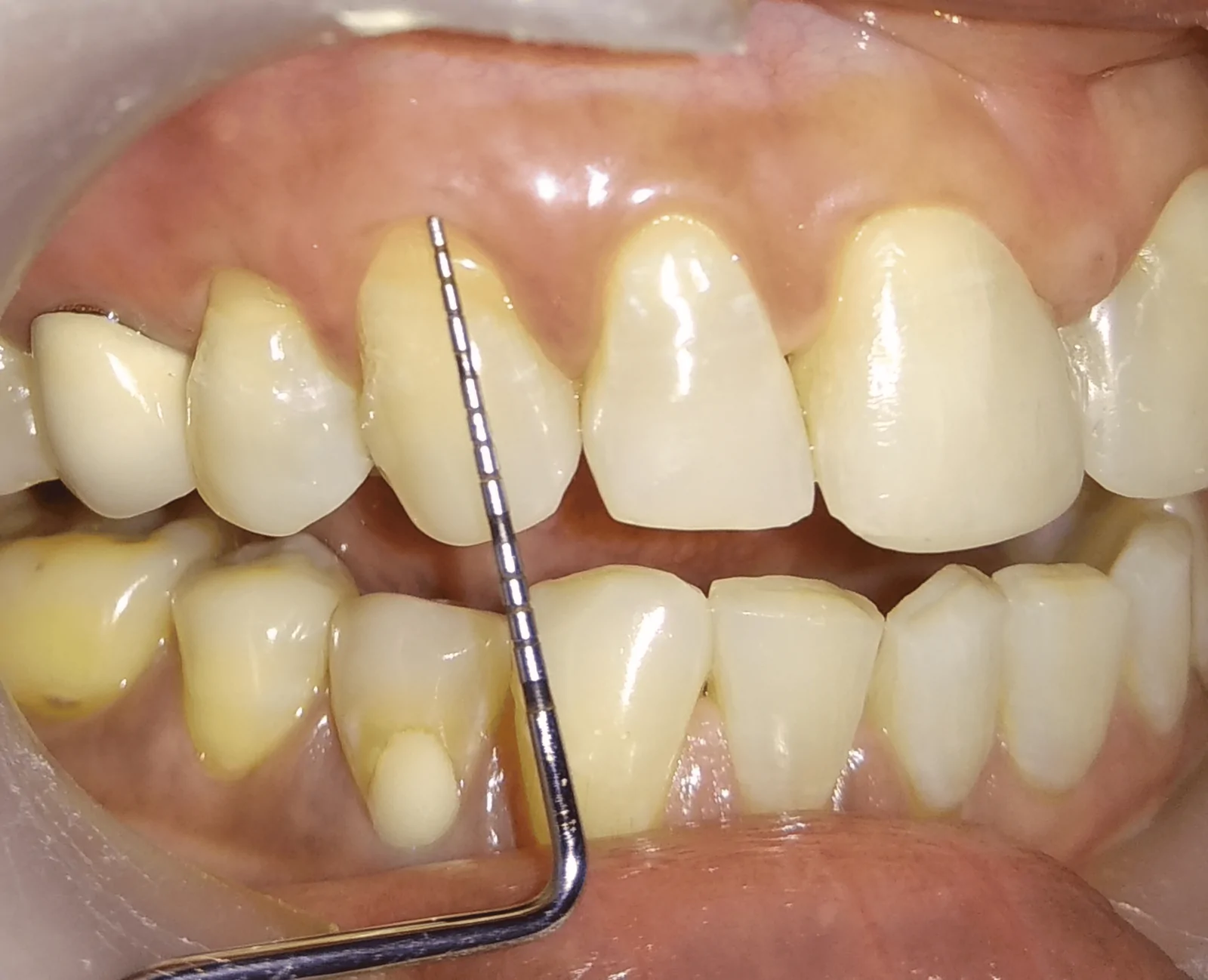
Measurement of recession using a UNC-15 periodontal probe for the coronally advanced flap (CAF) procedure

**Figure 2:**
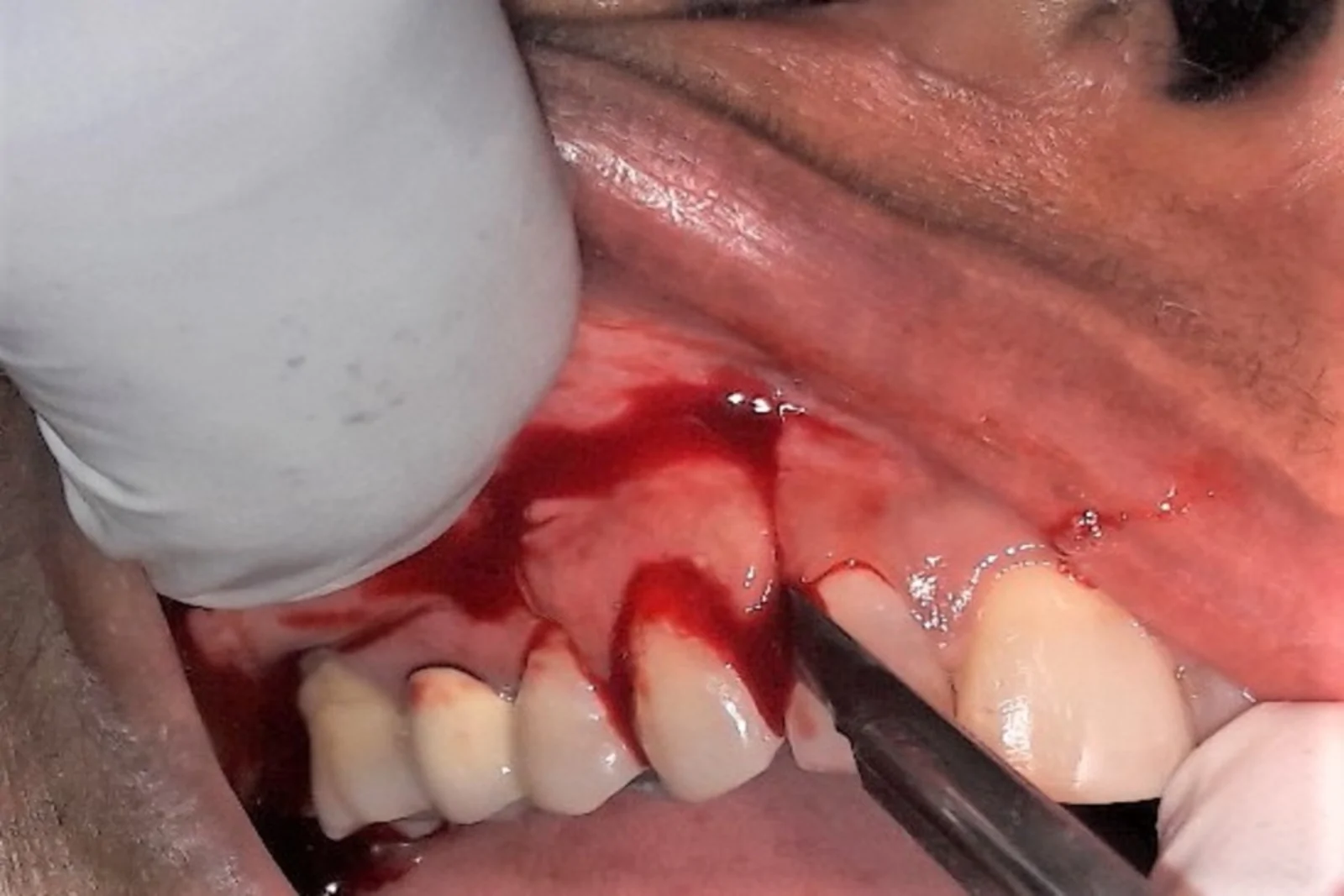
Incision given for the coronally advanced flap (CAF) procedure

**Figure 3:**
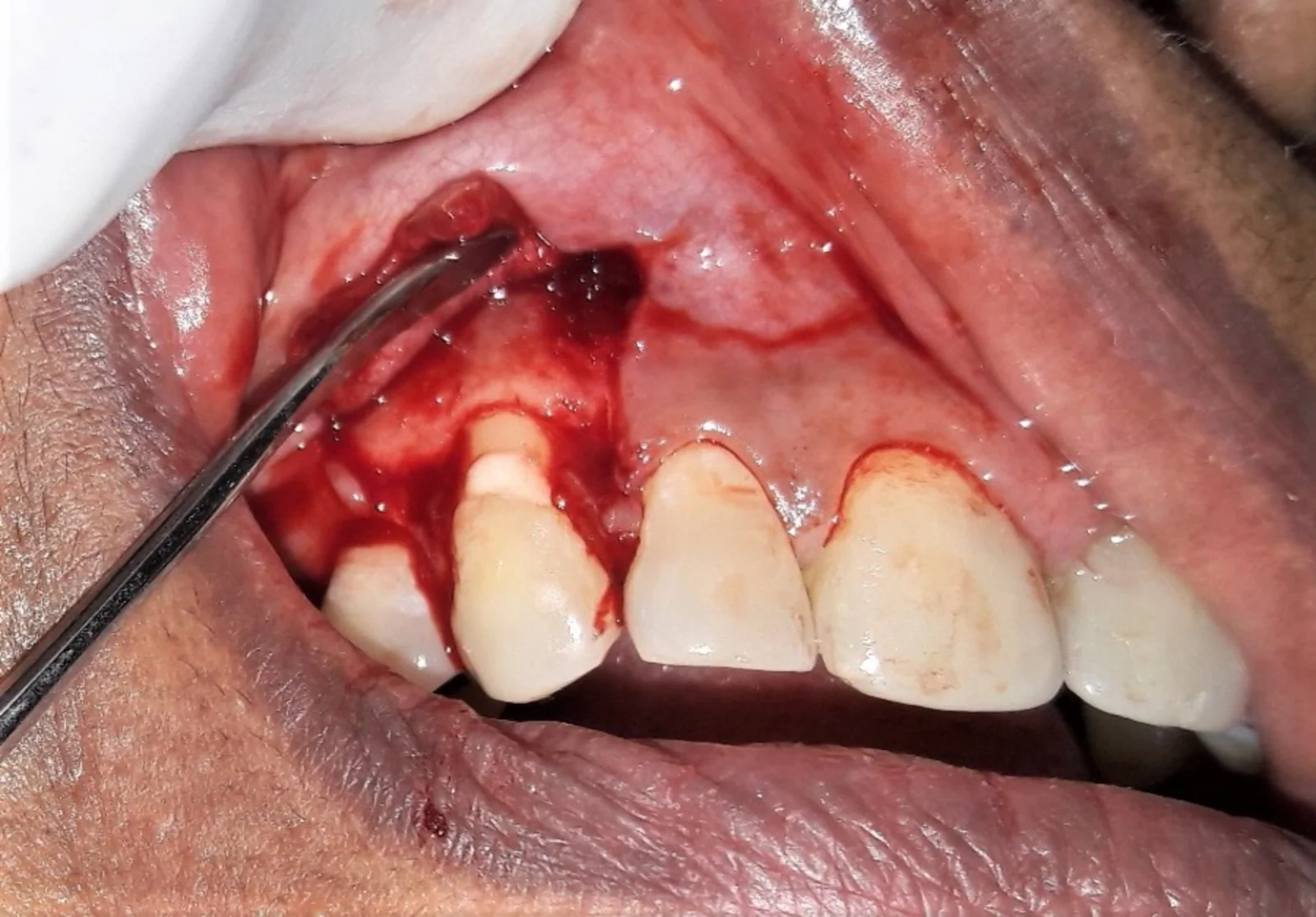
Split-full-split thickness flap reflected

**Figure 4:**
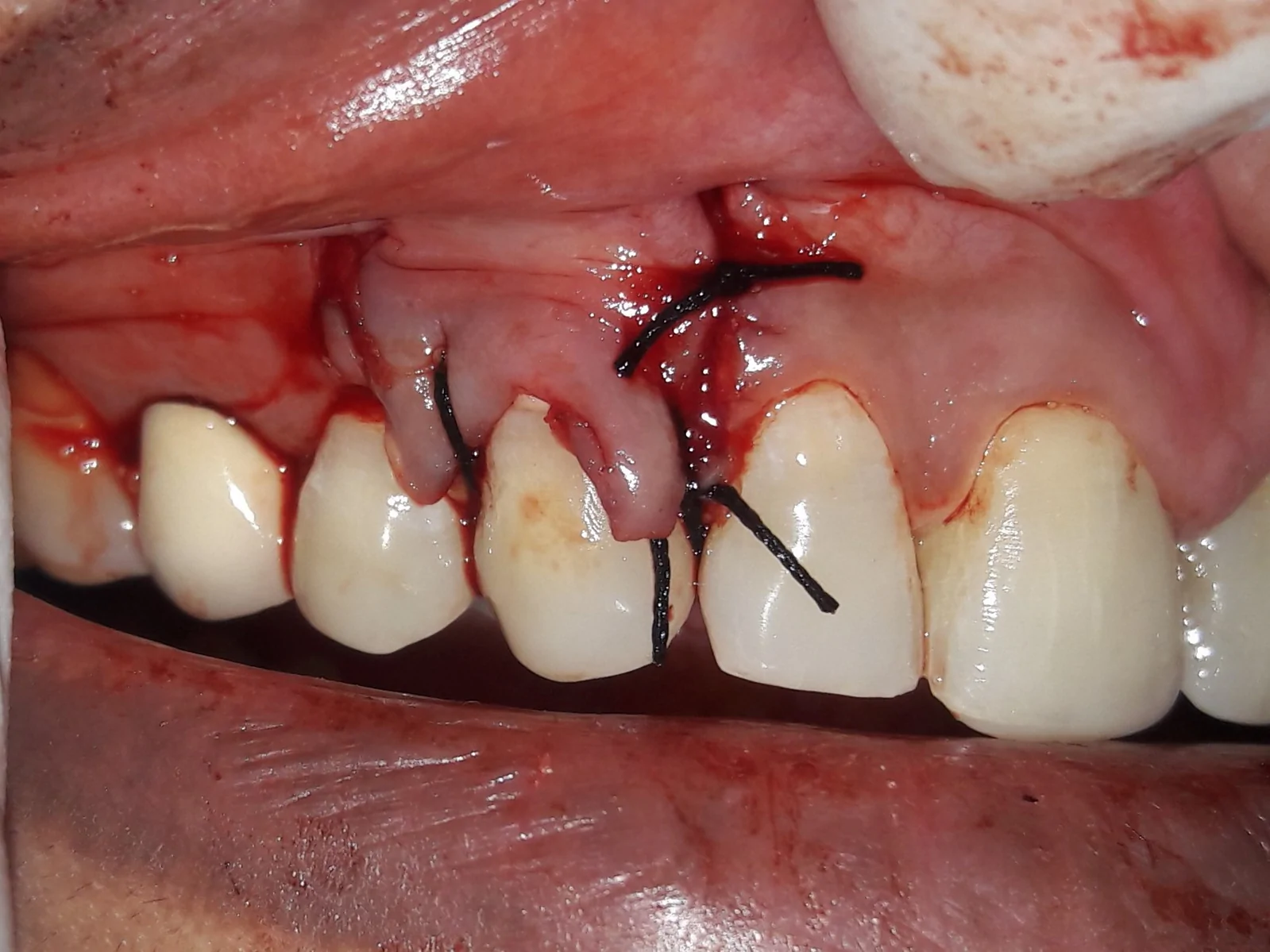
Flap coronally advanced and sutured

**Figure 5:**
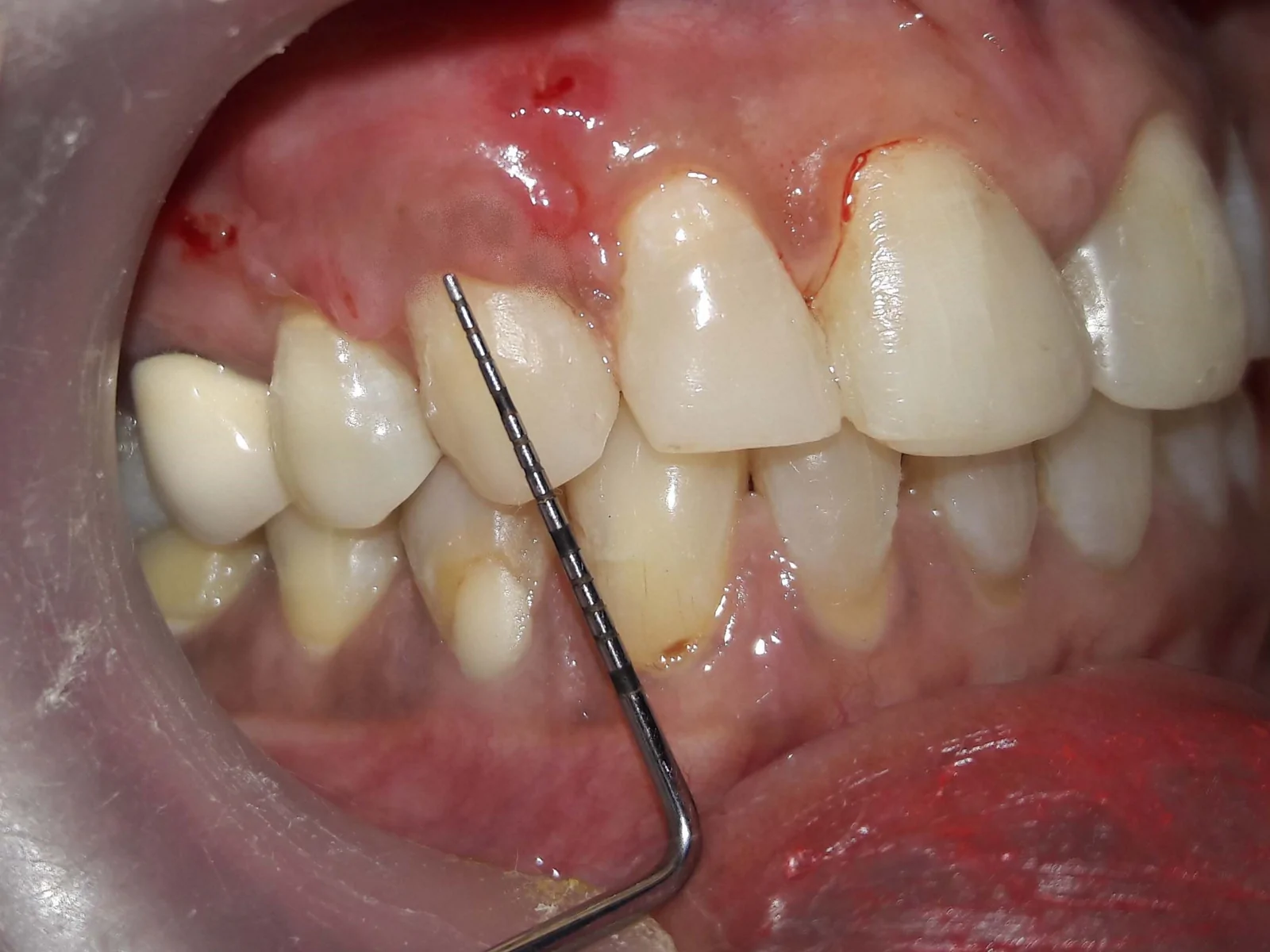
Two-week follow-up (Group A)

**Figure 6:**
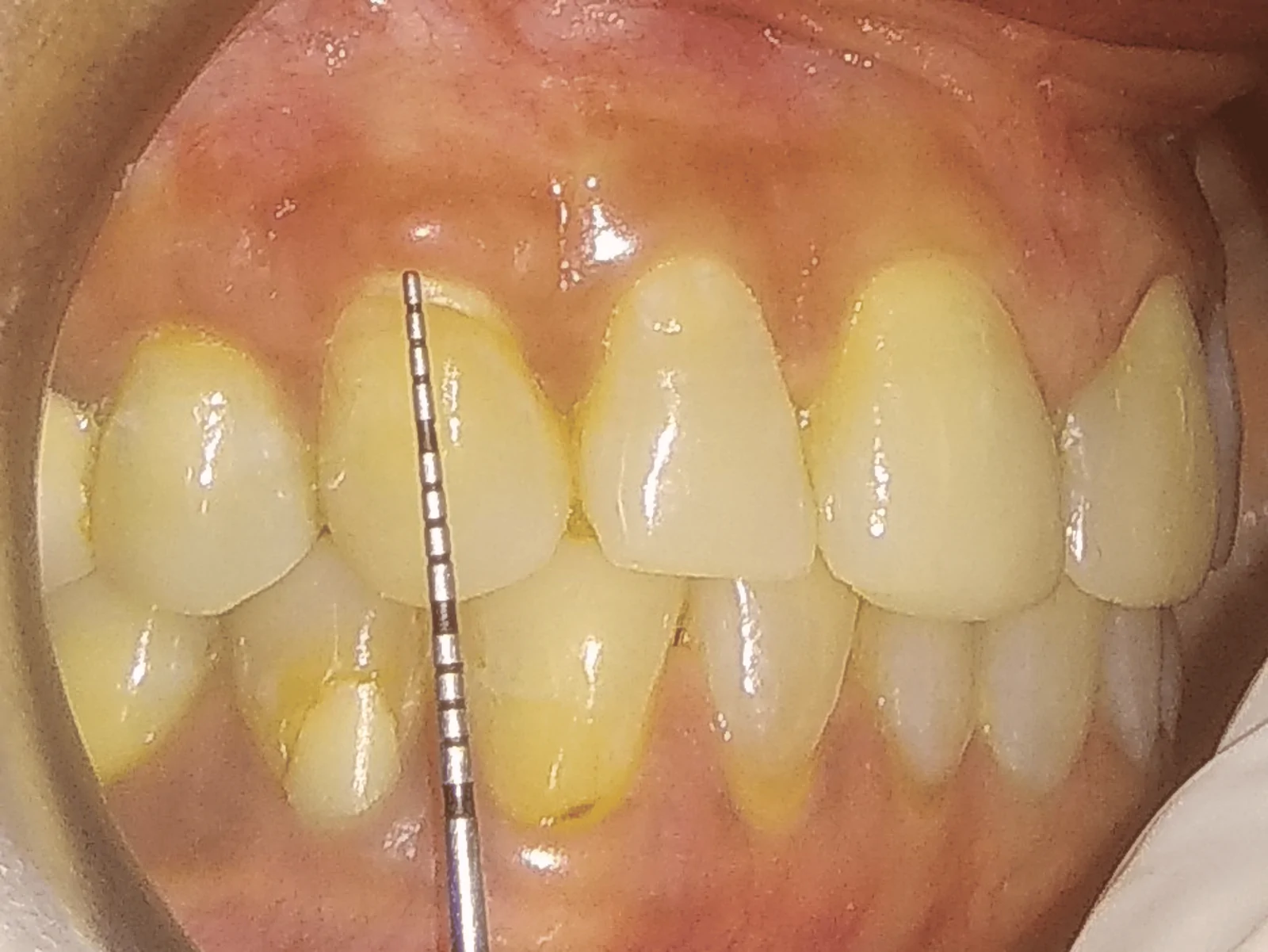
Three-month follow-up (Group A)

**Figure 7:**
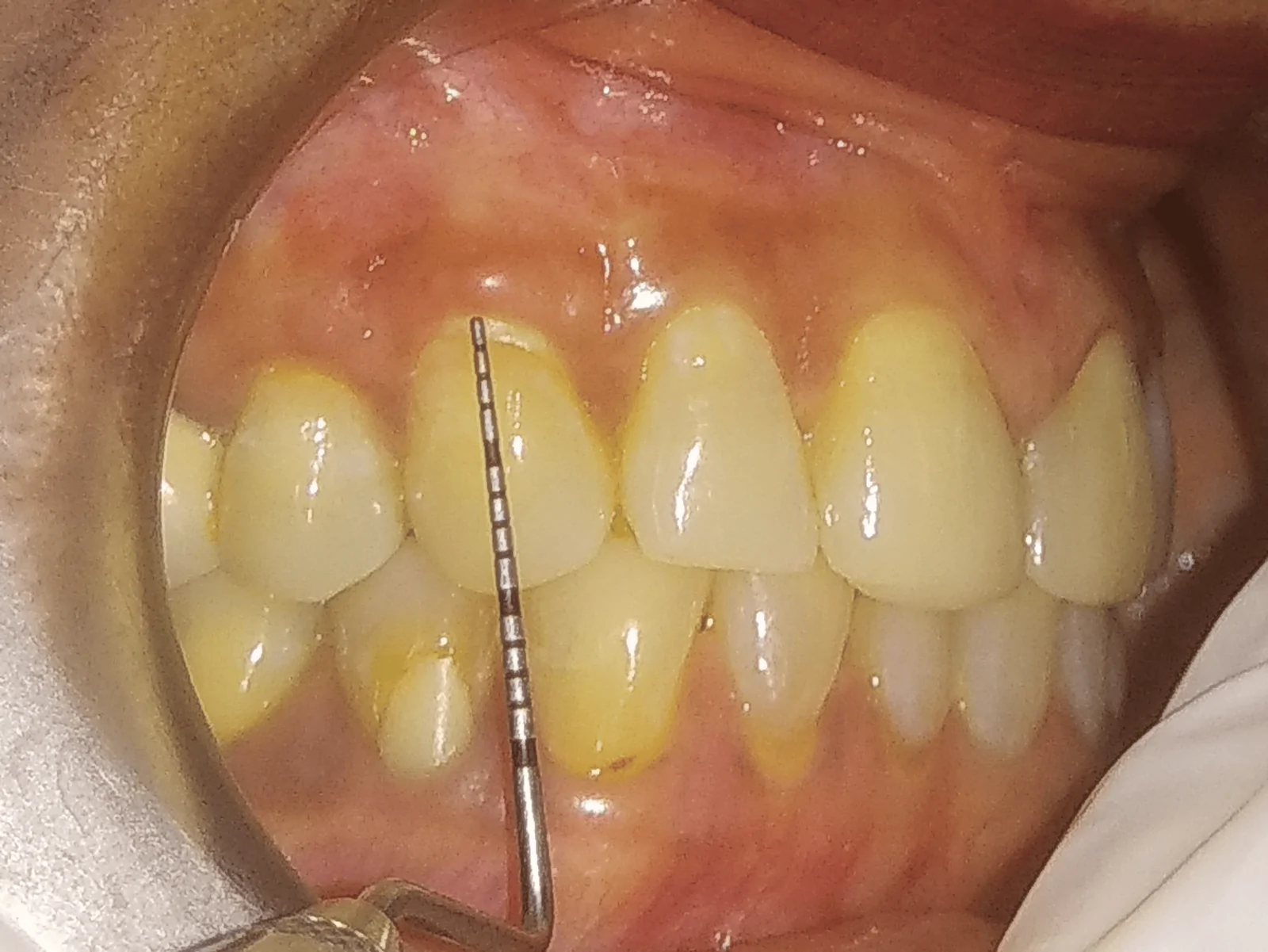
Six-month follow-up (Group A)

The CAF procedure was performed according to the technique described by De Sanctis and Zucchelli [9]. A trapezoidal split-full-split thickness flap was raised through an intrasulcular incision with two horizontal incisions at the interdental papilla and two oblique vertical releasing incisions extending beyond the mucogingival junction. Subsequently, adjacent papillae were de-epithelialized, and the flap was advanced coronally and stabilized at least 1 mm coronal to the CEJ using sling and interrupted sutures. Finally, a periodontal dressing was applied, which was removed after 14 days (Figures 1-7).

Semilunar coronally repositioned flap procedure

**Figure 8:**
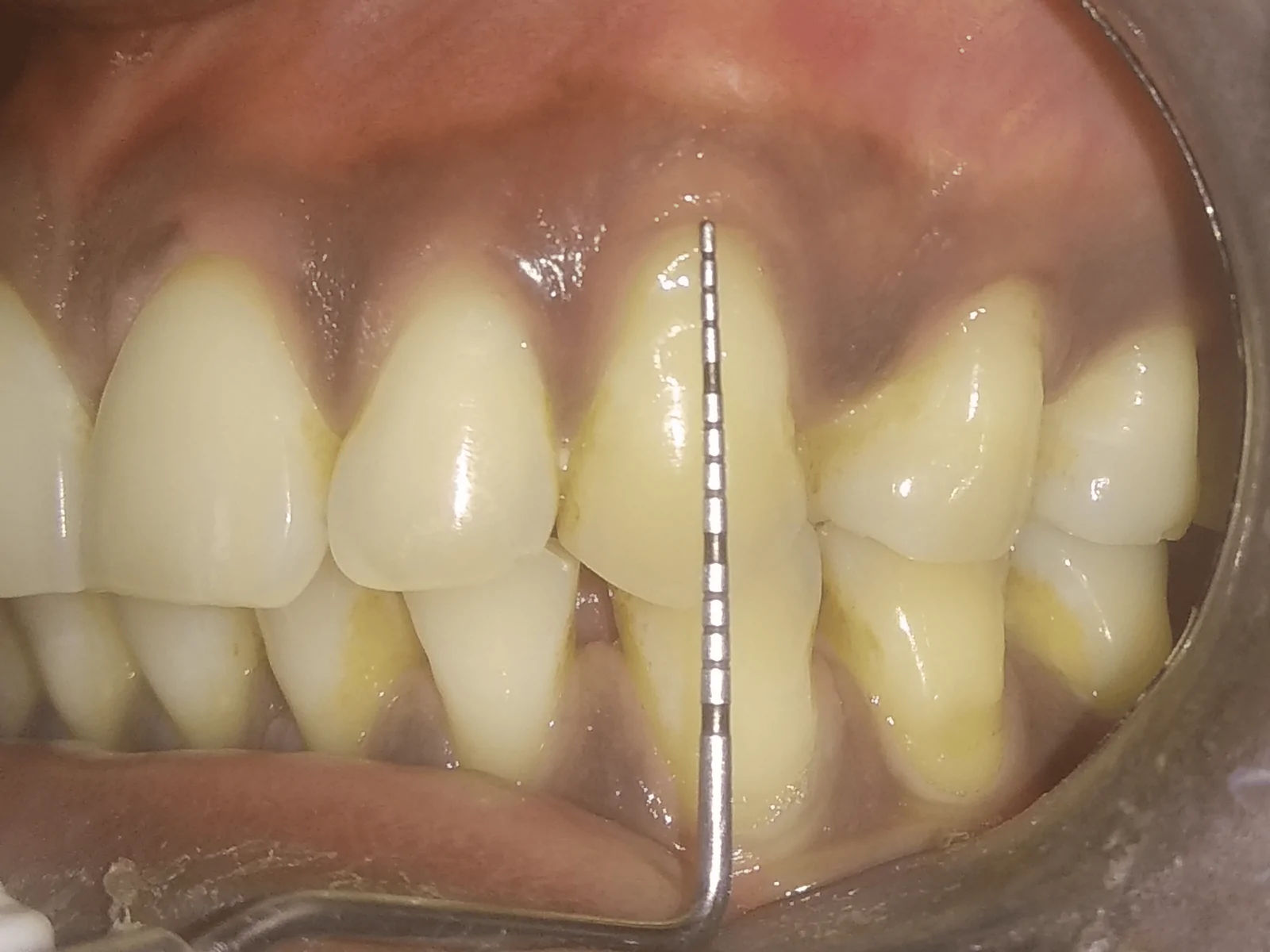
Measurement of recession using a UNC-15 periodontal probe for the semilunar coronally repositioned flap (SCRF) procedure

**Figure 9:**
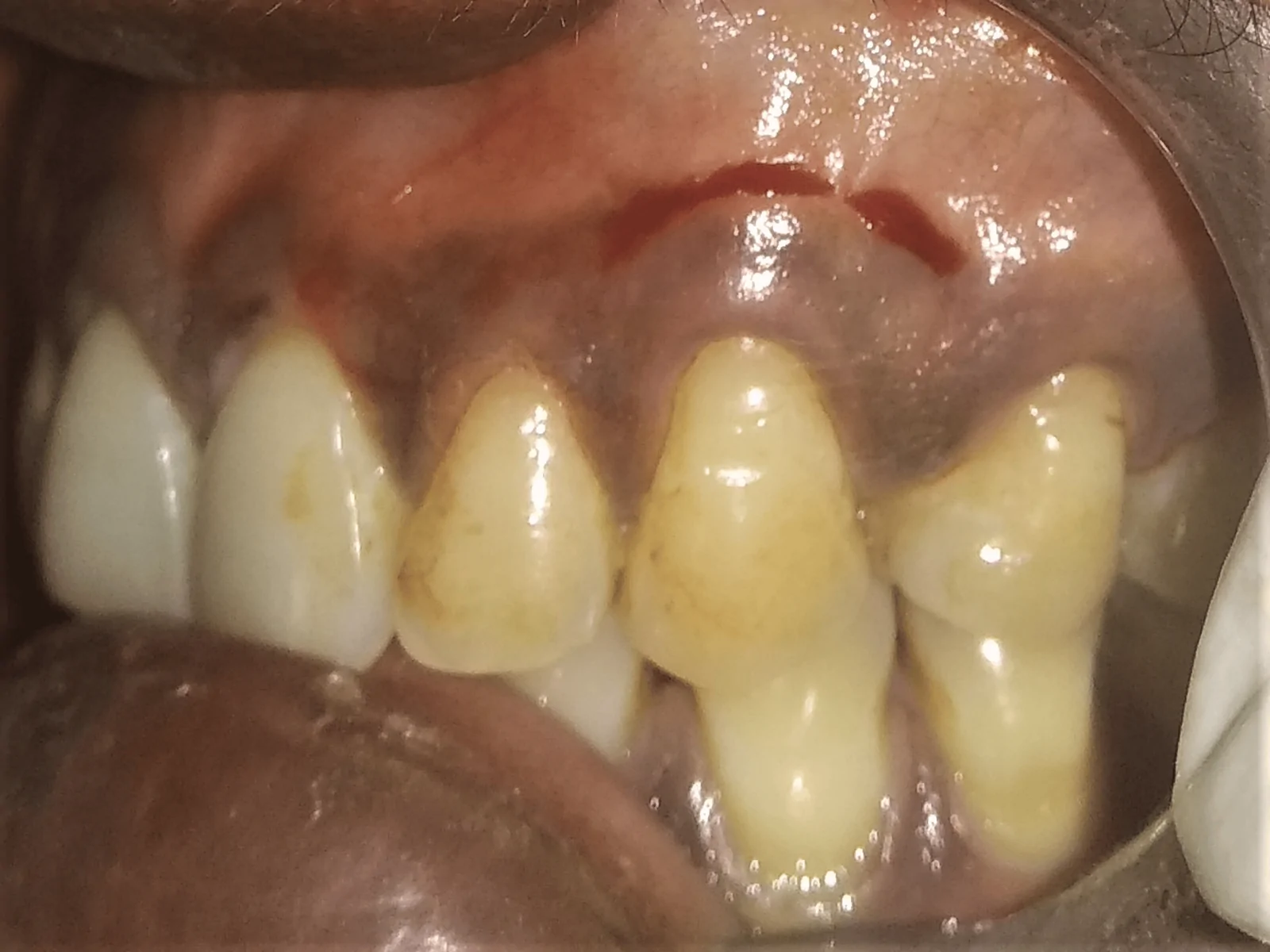
Incision given for the semilunar coronally repositioned flap (SCRF) procedure

**Figure 10:**
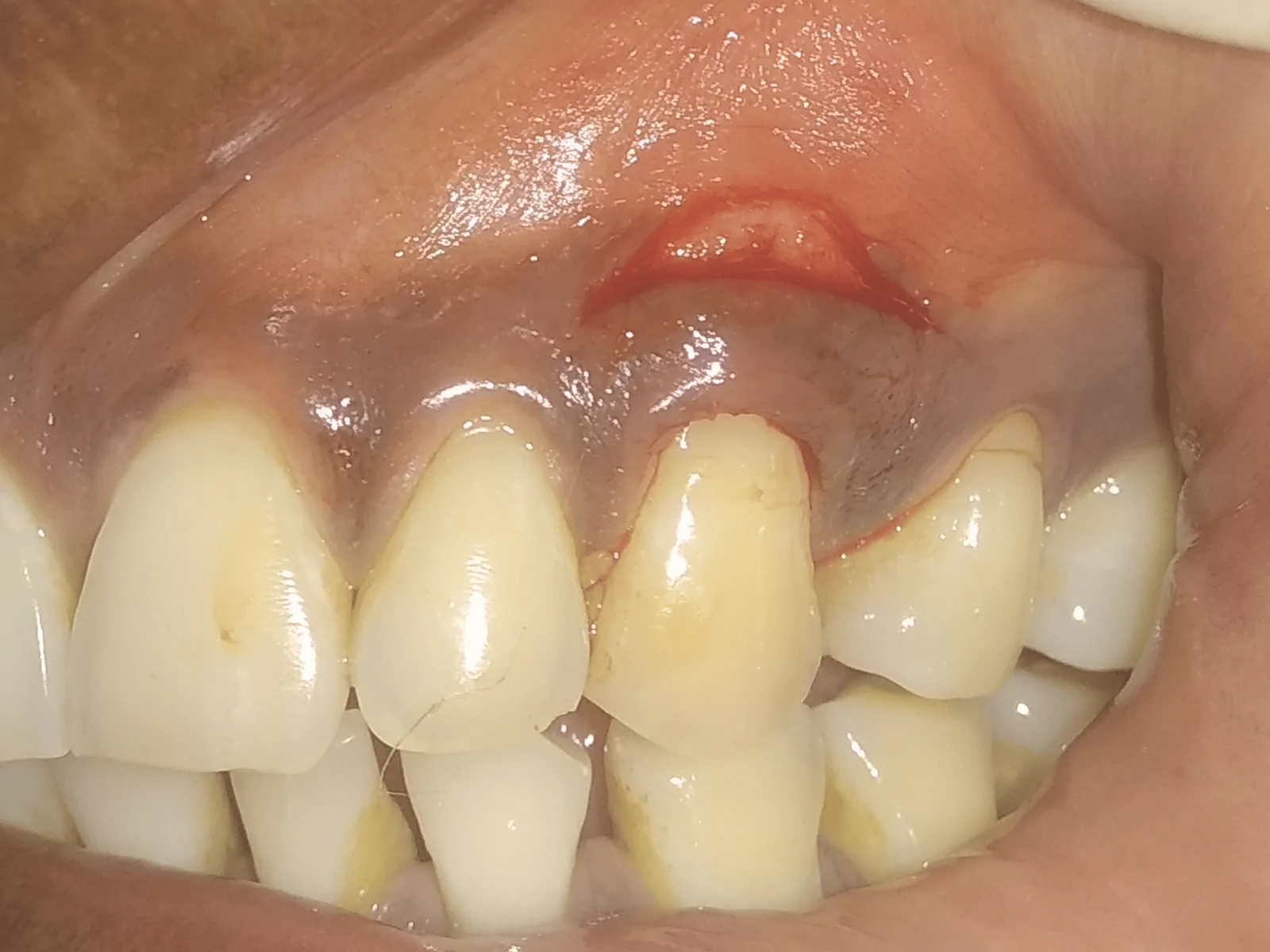
Semilunar coronally repositioned flap

**Figure 11:**
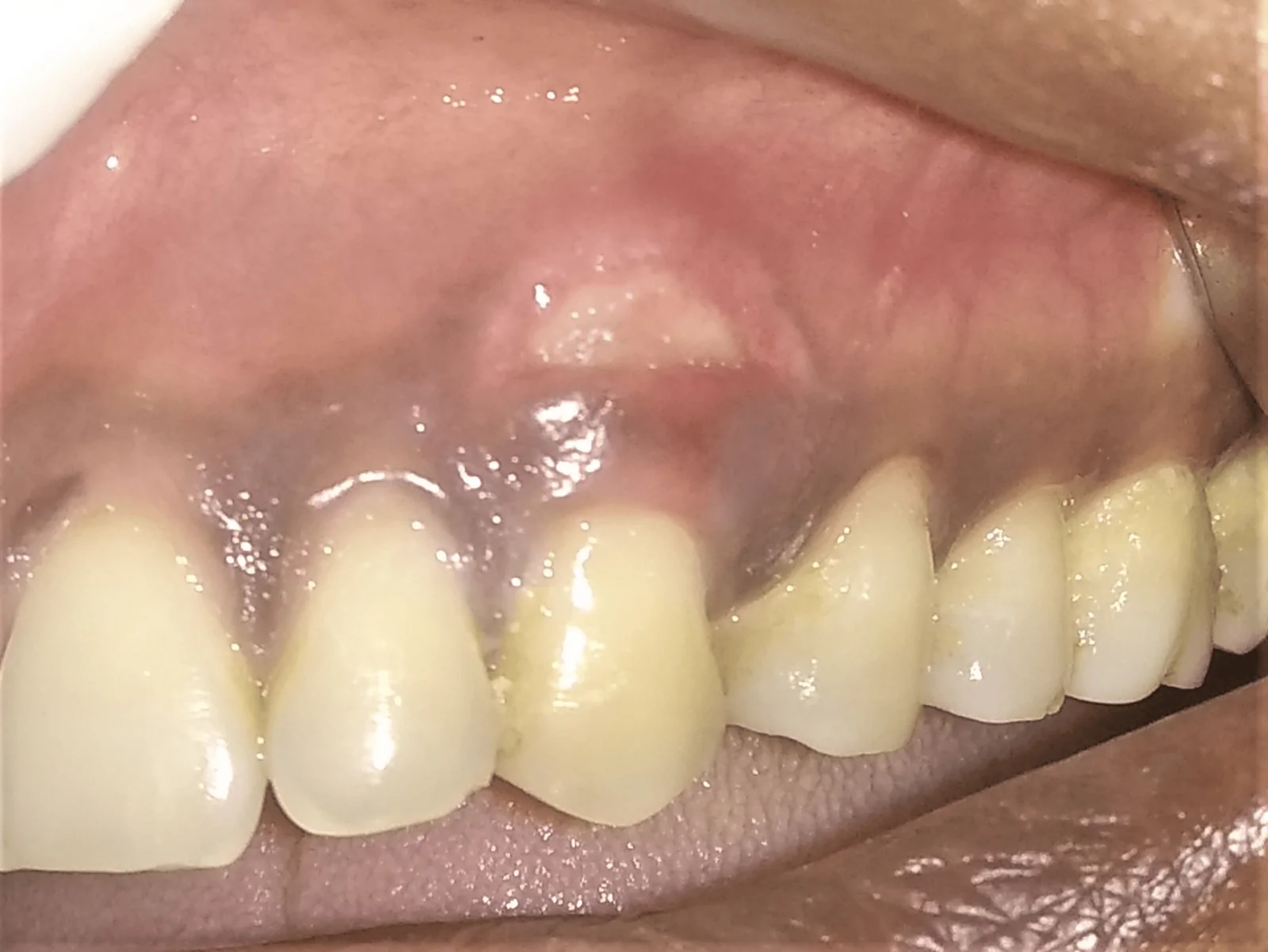
Two-week follow-up (Group B)

**Figure 12:**
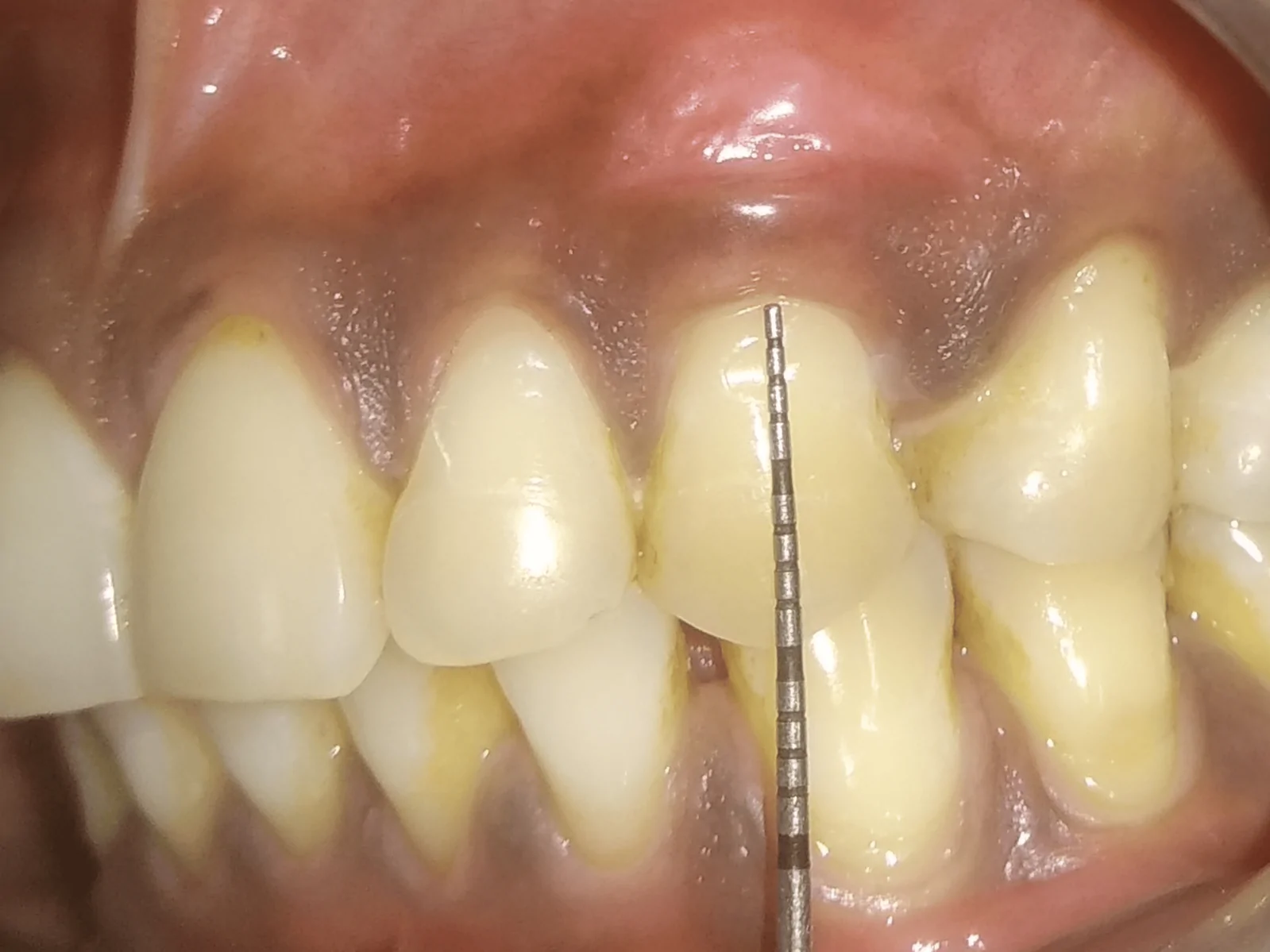
Three-month follow-up (Group B)

**Figure 13:**
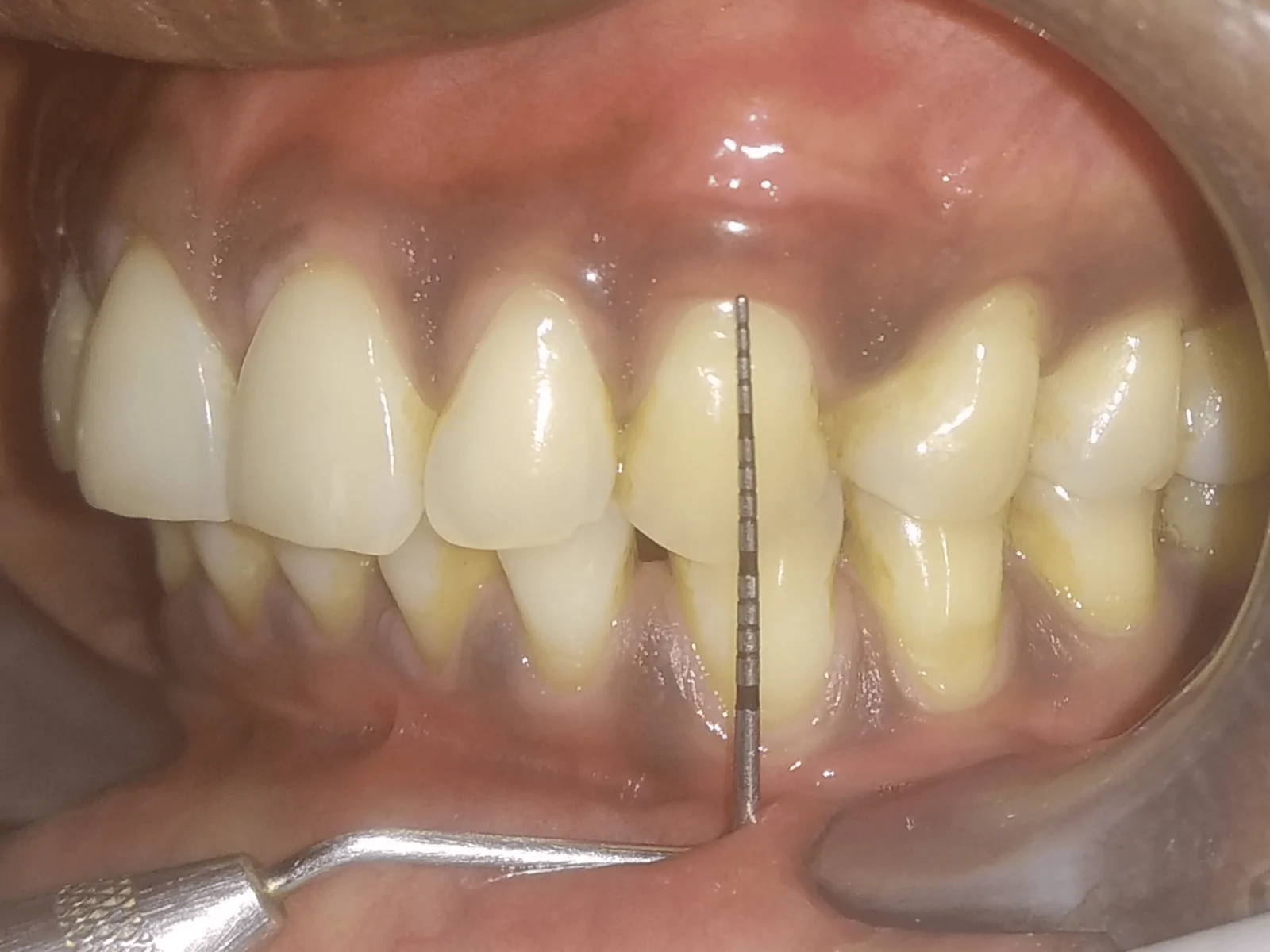
Six-month follow-up (Group B)

The SCRF procedure was performed according to the technique described by Tarnow [5]. A semilunar incision was placed in the attached gingiva parallel to the gingival margin while preserving the interdental papillae. Thereafter, a split-thickness dissection was carried out coronally to connect it with the intrasulcular incision. The flap was passively repositioned coronally to the CEJ and stabilized with gentle pressure using a moist gauze for five minutes. No sutures were placed. At the end, a periodontal dressing was placed, which was removed after 14 days (Figures 8-13).

All 20 patients completed the study and were available for evaluation at baseline, three months, and six months. Healing was uneventful in both groups, with no postoperative complications such as bleeding, suppuration, infection, swelling, or persistent discomfort.

Following data collection, all the data were entered into a master chart and analyzed using Statistical Package for the Social Sciences (SPSS) software version 24.0 (IBM Corp., Armonk, NY). Continuous variables were presented as mean ± standard deviation (SD). Normality of data was tested by the Kolmogorov-Smirnov test. Intergroup comparisons were performed using the Student's unpaired t-test/Mann-Whitney U test. Intragroup comparisons between different time intervals were performed using the Student's paired t-test/Wilcoxon signed-rank test, depending on data distribution. Throughout the analysis, p-value < 0.05 was considered significant at the 95% confidence level.

## Results

Recession height

**Figure 14:**
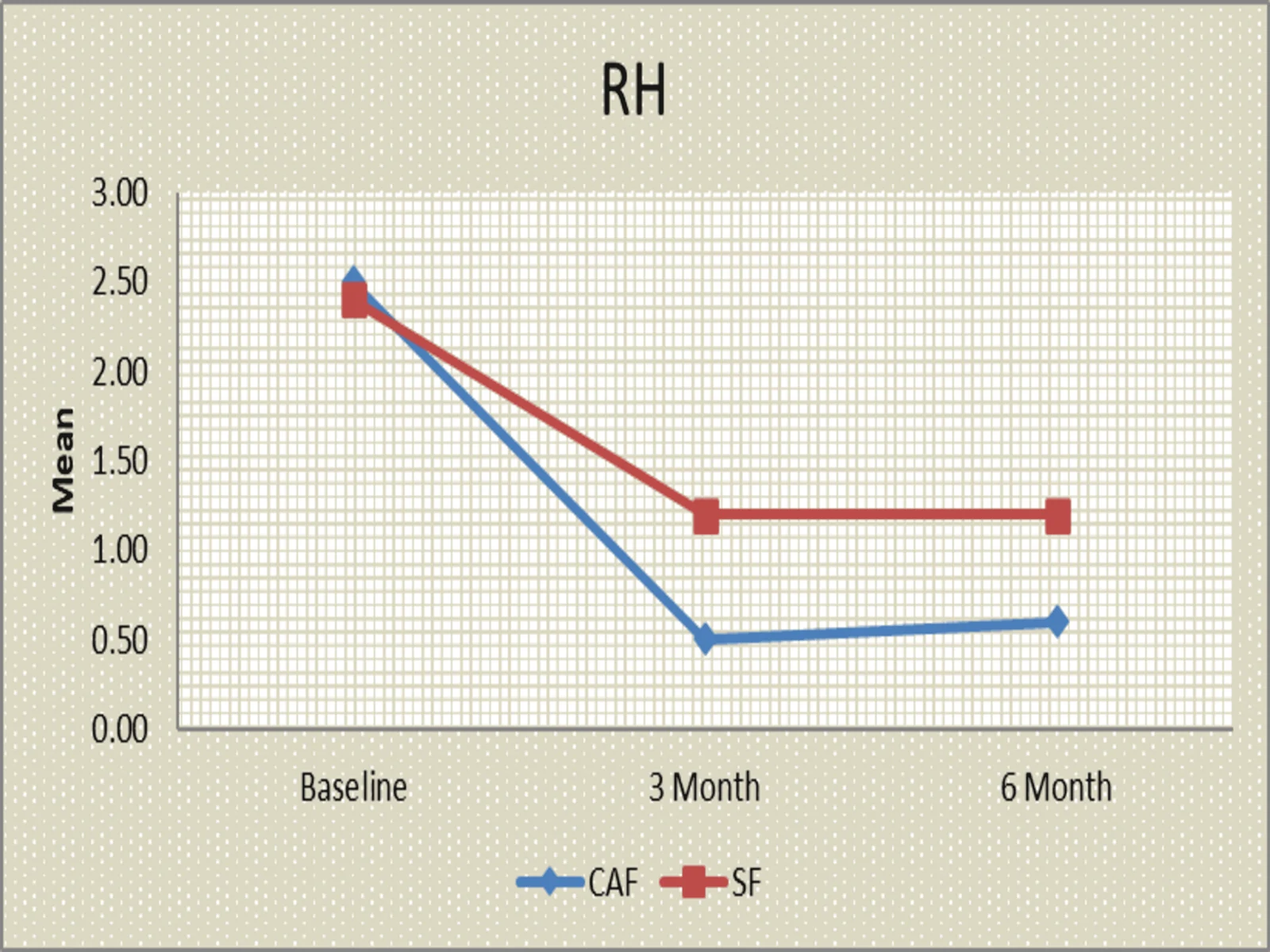
Line graph of recession height CAF, coronally advanced flap; RH, recession height; SF, semilunar flap

Both treatment modalities resulted in significant reductions in RH from baseline to three and six months (Figure 14). At baseline, mean RH was comparable between the groups. However, at six months, RH was significantly lower in the CAF group compared with the SCRF group(p < 0.05) (Table 1). Furthermore, intragroup analysis demonstrated statistically significant reductions in RH from baseline to three months and baseline to six months in both groups (p < 0.001). No significant changes were observed between three and six months (Table 2).

**Table 1:** Intergroup comparison of recession height Student's unpaired t-test; p < 0.05 = statistically significant.

Time interval	Group A: mean ± SD (mm)	Group B: mean ± SD (mm)	t-value	p-value
Baseline	2.50 ± 0.53	2.40 ± 0.52	0.43	0.67
Three months	0.50 ± 0.53	1.20 ± 0.63	2.69	0.015
Six months	0.60 ± 0.52	1.20 ± 0.63	2.32	0.032

**Table 2:** Intragroup comparison of recession height at different time intervals Student's paired t-test; p < 0.05 = statistically significant.

Group	Baseline to three months	Baseline to six months	Three months to six months
Mean difference			
A	2	1.9	0.1
B	1.2	1.2	0
t-value			
A	13.42	19	1
B	9	9	NA
p-value			
A	<0.001	<0.001	0.343
B	<0.001	<0.001	NA

Probing depth

**Figure 15:**
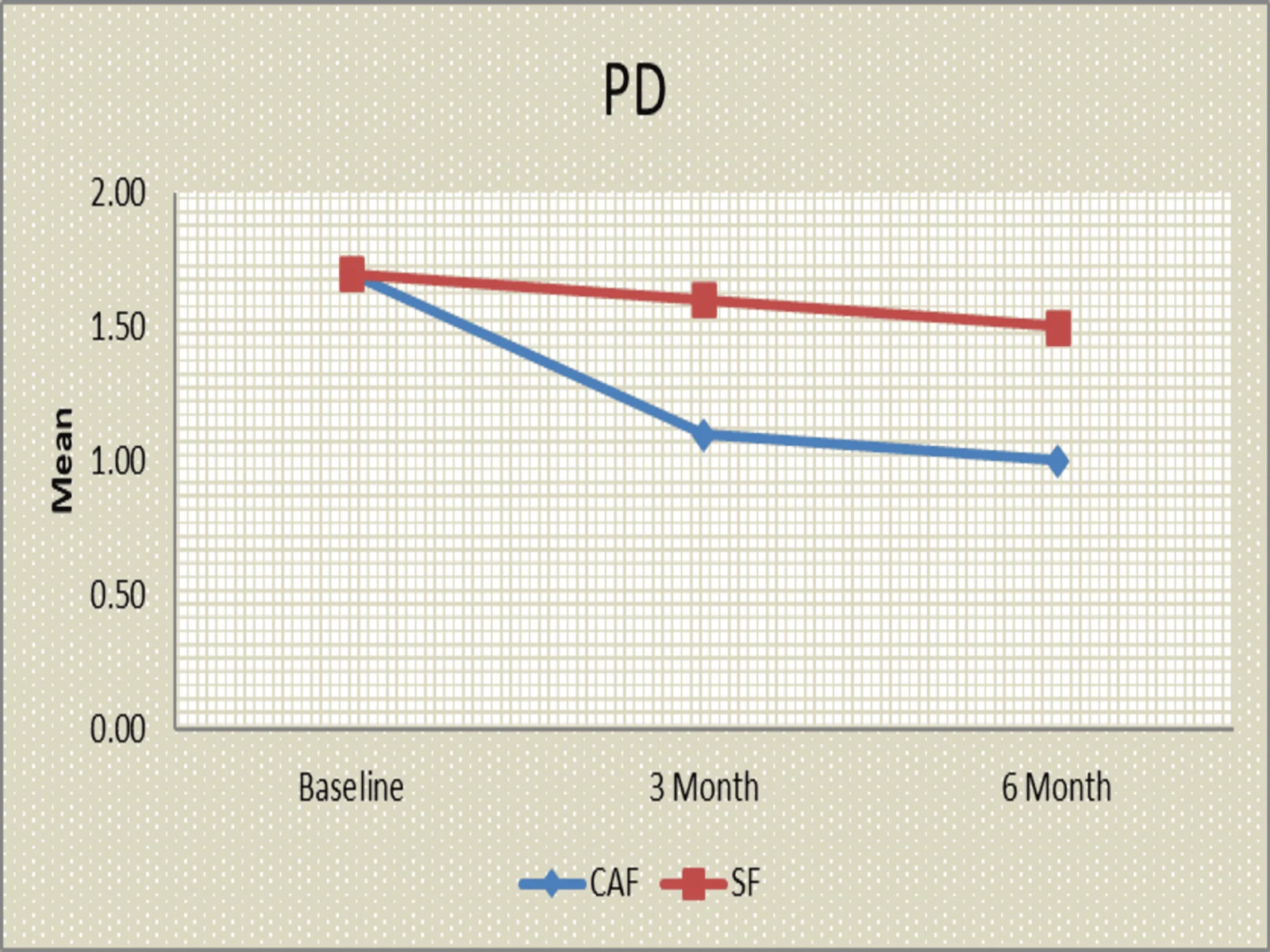
Line graph for probing depth PD, probing depth; RH, recession height; SF, semilunar flap

The mean PD at baseline was identical in both groups. At six months, PD was reduced to 1.0 ± 0.0 mm in the CAF group and 1.5 ± 0.53 mm in the SCRF group (Figure 15). Also, intergroup comparison revealed significantly lower PD values in the CAF group at both three and six months (p < 0.05) (Table 3). Intragroup analysis demonstrated significant reductions in PD in the CAF group between baseline and follow-up periods (p < 0.05), whereas changes in the SCRF group were not statistically significant (Table 4).

**Table 3:** Intergroup comparison of probing depth Student's unpaired t-test; p < 0.05 = statistically significant.

Time interval	Group A (mean ± SD)	Group B (mean ± SD)	t-value	p-value
Baseline	1.70 ± 0.48	1.70 ± 0.48	0	1
Three months	1.10 ± 0.32	1.60 ± 0.52	2.61	0.018
Six months	1.00 ± 0.00	1.50 ± 0.53	3	0.018

**Table 4:** Intragroup comparison of probing depth at different time intervals Student's paired t-test; p < 0.005 = statistically significant.

Group	Baseline to three months	Baseline to six months	Three months to six months
Mean difference			
A	0.6	0.7	0.1
B	0.1	0.2	0.1
t-value			
A	3.67	4.58	1
B	1	1.5	1
p-value			
A	0.005	0.001	0.343
B	0.343	0.168	0.343

Clinical attachment level

**Figure 16:**
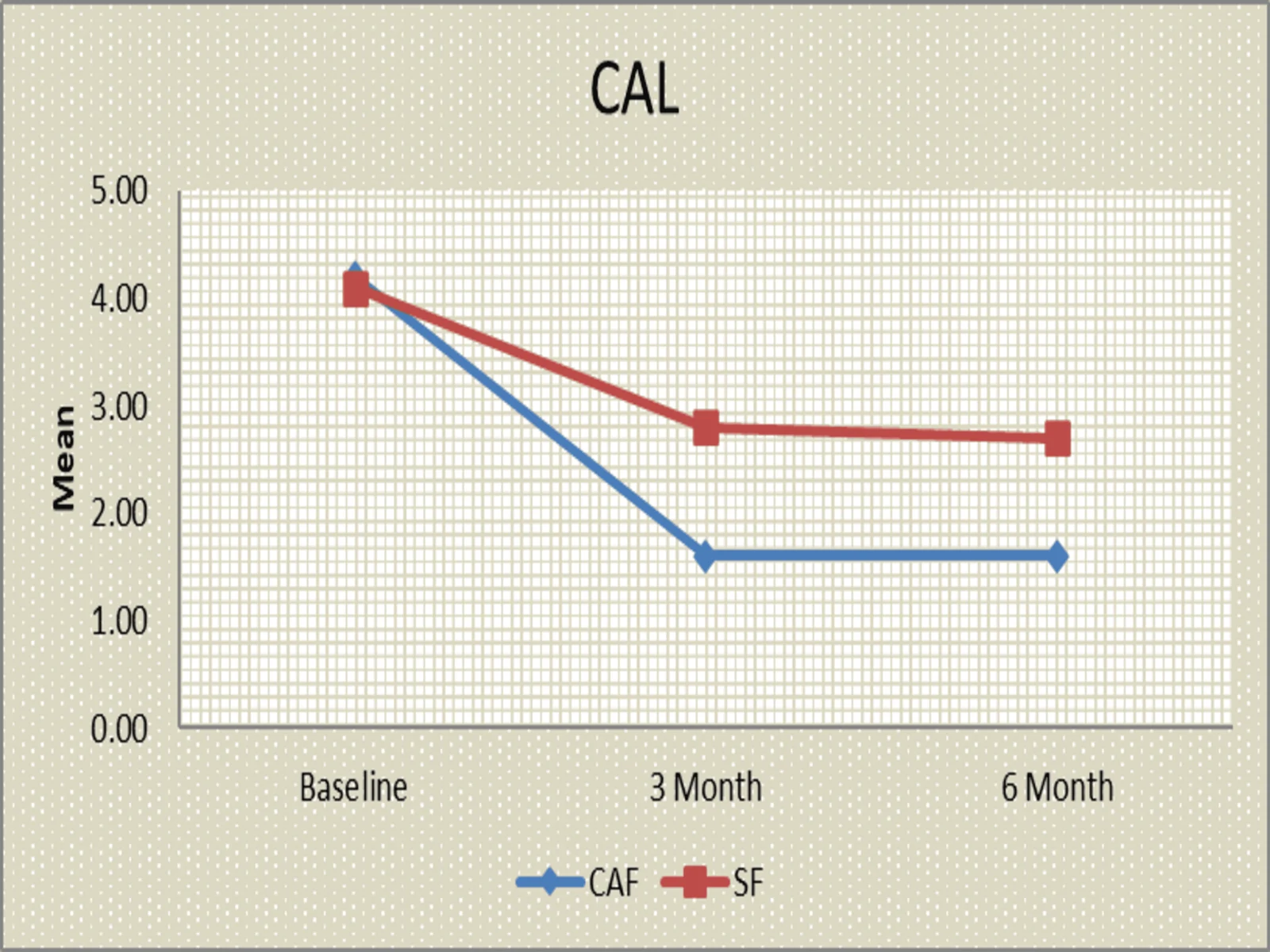
Line graph of clinical attachment level CAL, clinical attachment level; RH, recession height; SF, semilunar flap

Both treatment modalities resulted in significant clinical attachment gain (Figure 16). At baseline, CAL values were comparable between groups. However, at six months, mean CAL was significantly lower in the CAF group than in the SCRF group (p < 0.001), implying more CAL gain in the CAF than in the SCRF group (Table 5). Intragroup comparisons showed significant improvements from baseline to both three and six months in both groups (p < 0.001) (Table 6).

**Table 5:** Intergroup comparison of clinical attachment level Student's unpaired t-test; p < 0.05 = statistically significant.

Time interval	Group A (mean ± SD)	Group B (mean ± SD)	t-value	p-value
Baseline	4.20 ± 0.63	4.10 ± 0.74	0.33	0.749
Three months	1.60 ± 0.52	2.80 ± 0.63	4.65	<0.001
Six months	1.60 ± 0.52	2.70 ± 0.48	4.92	<0.001

**Table 6:** Intragroup comparison of clinical attachment level at different time intervals Student's paired t-test; p < 0.05 = statistically significant.

Group	Baseline to three months	Baseline to six months	Three months to six months
Mean difference			
A	2.6	2.6	0
B	1.3	1.4	0.1
t-value			
A	15.92	15.92	NA
B	9	8.57	1
p-value			
A	<0.001	<0.001	NA
B	<0.001	<0.001	0.343

Width of keratinized gingiva

**Figure 17:**
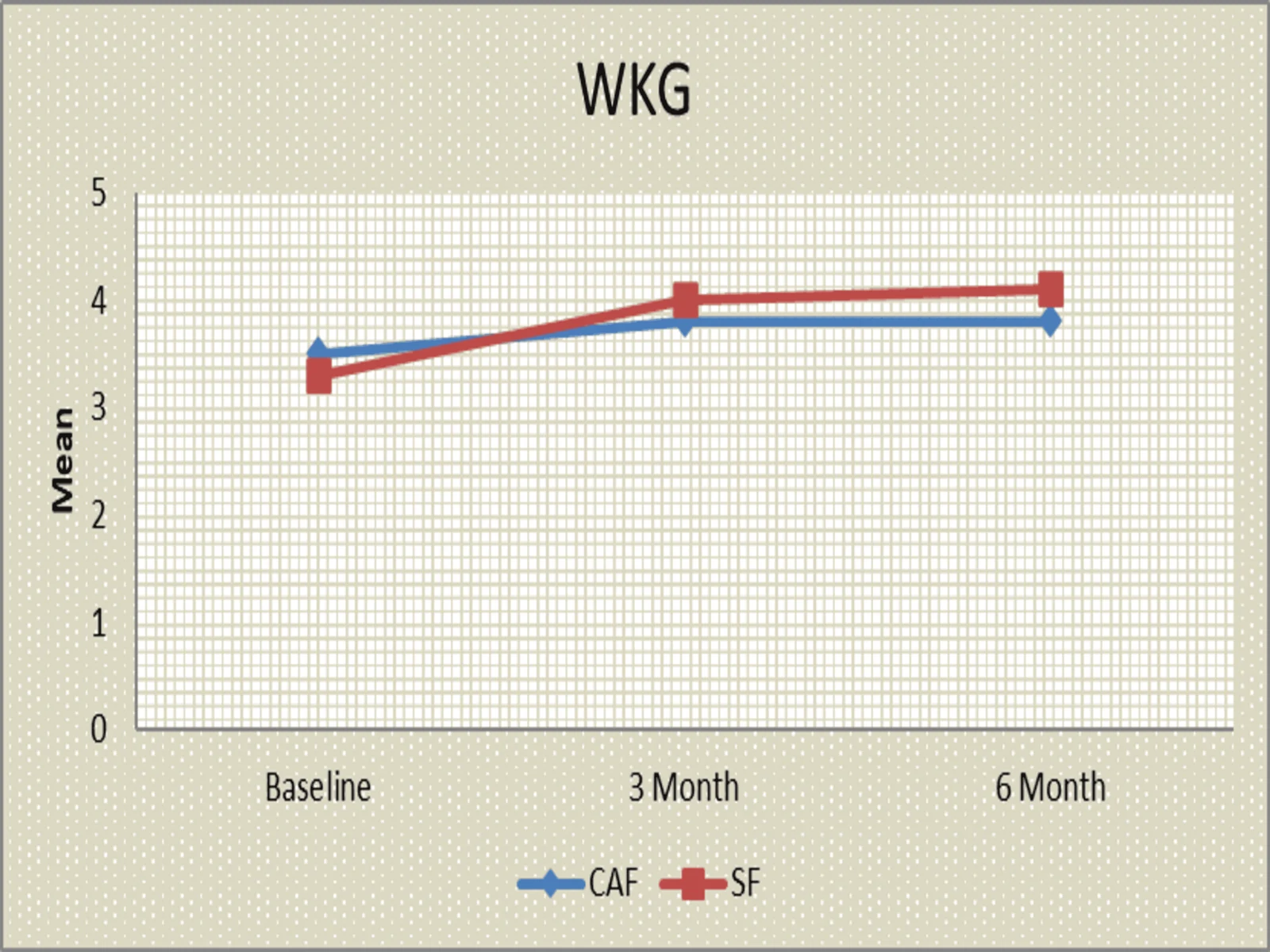
Line graph for width of keratinized gingiva RH, recession height; SF, semilunar flap; WKG, width of keratinized gingiva

An increase in WKG was observed in both treatment groups (Figure 17). Baseline values were 3.50 ± 0.53 mm for CAF and 3.30 ± 0.48 mm for SCRF. By the end of six months, WKG increased to 3.80 ± 0.79 mm in the CAF group and 4.10 ± 0.74 mm in the SCRF group. Although the increase was greater in the SCRF group, the intergroup difference was not statistically significant (Table 7). Furthermore, intragroup analysis revealed a statistically significant increase in WKG in the SCRF group (p < 0.001), whereas changes observed in the CAF group were not statistically significant (Table 8).

**Table 7:** Intergroup comparison of width of keratinized gingiva Student's unpaired t-test; p < 0.05 = statistically significant.

Time interval	Group A (mean ± SD)	Group B (mean ± SD)	t-value	p-value
Baseline	3.50 ± 0.53	3.30 ± 0.48	0.89	0.388
Three months	3.80 ± 0.79	4.00 ± 0.67	0.61	0.548
Six months	3.80 ± 0.79	4.10 ± 0.74	0.88	0.391

**Table 8:** Intragroup comparison of width of keratinized gingiva at different time intervals Student's paired t-test; p < 0.05 = statistically significant.

Group	Baseline to three months	Baseline to six months	Three months to six months
Mean difference			
A	0.3	0.3	0
B	0.7	0.8	0.1
t-value			
A	-1.41	-1.41	NA
B	3	4	1
p-value			
A	0.193	0.193	NA
B	0.01	0.003	0.343

Root coverage percentage

At six months, the mean percentage of root coverage was 78.33% in the CAF group and 53.33% in the SCRF group. In the CAF group, five out of 10 sites achieved greater than 90% root coverage, three sites achieved 50-90% coverage, and two sites achieved 30-50% coverage. In contrast, in the SCRF group, only two out of 10 sites achieved greater than 90% root coverage, while eight sites demonstrated 30-50% coverage. Overall, the CAF demonstrated better clinical outcomes with respect to recession reduction, root coverage percentage, and gain in CAL, whereas SCRF showed a greater increase in the WKG.

## Discussion

The primary objective of the present randomized clinical study was to compare the clinical efficacy of CAF and SCRF in the treatment of Miller's class I gingival recession defects in maxillary anterior teeth.

Complete root coverage is considered to be an ideal treatment outcome of root coverage therapy because not only does it restore aesthetics but also reduces dentinal hypersensitivity and lowers the risk of root caries [10]. Thus, the root coverage that does not reach the CEJ may not be satisfactory to the patients [11]. In the present study, CAF achieved a mean root coverage of 78.33%, whereas SCRF achieved 53.33%. Moreover, complete root coverage of more than 90% was observed in 50% of CAF-treated sites compared with 20% of SCRF-treated sites. These clinical findings are consistent with a study done by Santana et al., who also found superior root coverage and a higher frequency of complete root coverage with CAF compared with SCRF [12]. Similarly, Moka et al. reported better root coverage and more predictable results with CAF [13].

The better performance of CAF might be attributed to several surgical factors. In CAF, vertical releasing incisions [8], adequate flap mobilization, and sling sutures facilitate precise coronal positioning and stabilization of the flap over the exposed root surface [14,15]. In contrast, the original SCRF technique relies primarily on passive flap adaptation and periodontal dressing for stabilization. Also, horizontal incision in the SCRF procedure may have led to flap contraction during the healing period, which may have compromised the final position of the gingival margin and reduced root coverage outcomes [16,17].

Both treatment modalities produced significant reductions in RH; however, the reduction was greater in the CAF group. Mean RH decreased from 2.5 ± 0.53 mm to 0.6 ± 0.52 mm in the CAF group, compared with a reduction from 2.4 ± 0.52 mm to 1.2 ± 0.63 mm in the SCRF group. These findings further support the greater predictability of CAF in achieving coronal displacement and long-term stabilization of the gingival margin.

There was a significant reduction in the PD in the CAF group, whereas only minimal changes were observed in the SCRF group. Since the baseline PDs were shallow in both groups, the clinical significance of these changes is likely limited. Nonetheless, the findings suggest both treatment modalities provide favorable periodontal stability.

CAL improved significantly in both groups. However, CAF demonstrated a greater gain in attachment (2.6 mm) compared with SCRF (1.4 mm). Similar findings have been reported by Modica et al. [18], Del Pizzo et al. [19], and Berlucchi et. al. [20], who observed substantial attachment gain following CAF procedures. The greater attachment gain observed with CAF is likely related to enhanced root coverage and improved soft-tissue adaptation to the root surface.

Regarding the WKG, an increase in the width was observed in both groups, but the change was statistically significant only in the SCRF group. The increase in keratinized tissue associated with SCRF has been reported previously and may be explained by healing and maturation of the repositioned tissues [21], as well as the natural tendency of the mucogingival junction to return toward its genetically predetermined position [22]. Although the gain in keratinized tissue was greater in the SCRF group, the difference between the groups was not statistically significant. Contemporary systematic reviews continue to identify keratinized tissue gain as an important secondary outcome following root-coverage procedures, although the magnitude of gain varies according to the surgical technique and use of adjunctive graft materials [3,23,24].

In terms of postoperative healing, all the patients experienced uneventful recovery. However, sites treated with SCRF exhibited prolonged tissue discoloration and occasional scar formation during and after the healing phase. Similar observations have been reported previously and may represent an aesthetic limitation of the technique [12]. In contrast, CAF-treated sites showed favorable tissue integration with minimal scarring and satisfactory color matching with adjacent tissues.

Overall, within the limitations of the present study, CAF demonstrated superior clinical outcomes compared with SCRF for the treatment of Miller's class I gingival recession defects. The improved root coverage, greater attachment gain, and higher frequency of complete root coverage suggest that CAF is a more predictable treatment option for isolated recession defects in the maxillary anterior region.

Limitations

The present study had limitations including its small sample size and short follow-up duration of six months. Furthermore, patient-reported outcomes such as aesthetic satisfaction and dentinal hypersensitivity were not evaluated. Therefore, future randomized controlled trials with larger sample sizes, longer follow-up periods, and assessment of patient-centered outcomes are recommended to confirm these findings.

## Conclusions

Based on the findings of the present study, both the CAF and the SCRF were effective in treating Miller's class I gingival recession defects. SCRF offered the advantages of a simple, less invasive, and sutureless surgical approach with a significant increase in the WKG. However, CAF demonstrated greater recession reduction, higher root coverage percentage, better clinical attachment gain, and a higher frequency of complete root coverage. Therefore, within the limitations of this study, CAF appears to be the more predictable treatment modality for the management of isolated Miller's class I gingival recession defects in maxillary anterior teeth.
